# Are model organisms representative for climate change research? Testing thermal tolerance in wild and laboratory zebrafish populations

**DOI:** 10.1093/conphys/coz036

**Published:** 2019-06-24

**Authors:** Rachael Morgan, Josefin Sundin, Mette H Finnøen, Gunnar Dresler, Marc Martínez Vendrell, Arpita Dey, Kripan Sarkar, Fredrik Jutfelt

**Affiliations:** 1 Department of Biology, Norwegian University of Science and Technology, Trondheim, Norway; 2 Department of Neuroscience, Uppsala University, Uppsala, Sweden; 3 Department of Biology, University of Barcelona, Barcelona, Spain; 4 Department of Zoology, University of North Bengal, Darjeeling, Siliguri, West Bengal, India; 5 Rainbow Ornamental Fish Farm, Baxipara, Raninagar, Mohitnagar, Jalpaiguri, West Bengal, India

**Keywords:** Acclimation, critical thermal maxima, CT_max_, *Danio rerio*, domestication

## Abstract

Model organisms can be useful for studying climate change impacts, but it is unclear whether domestication to laboratory conditions has altered their thermal tolerance and therefore how representative of wild populations they are. Zebrafish in the wild live in fluctuating thermal environments that potentially reach harmful temperatures. In the laboratory, zebrafish have gone through four decades of domestication and adaptation to stable optimal temperatures with few thermal extremes. If maintaining thermal tolerance is costly or if genetic traits promoting laboratory fitness at optimal temperature differ from genetic traits for high thermal tolerance, the thermal tolerance of laboratory zebrafish could be hypothesized to be lower than that of wild zebrafish. Furthermore, very little is known about the thermal environment of wild zebrafish and how close to their thermal limits they live. Here, we compared the acute upper thermal tolerance (critical thermal maxima; CT_max_) of wild zebrafish measured on-site in West Bengal, India, to zebrafish at three laboratory acclimation/domestication levels: wild-caught, F_1_ generation wild-caught and domesticated laboratory AB-WT line. We found that in the wild, CT_max_ increased with increasing site temperature. Yet at the warmest site, zebrafish lived very close to their thermal limit, suggesting that they may currently encounter lethal temperatures. In the laboratory, acclimation temperature appeared to have a stronger effect on CT_max_ than it did in the wild. The fish in the wild also had a 0.85–1.01°C lower CT_max_ compared to all laboratory populations. This difference between laboratory-held and wild populations shows that environmental conditions can affect zebrafish’s thermal tolerance. However, there was no difference in CT_max_ between the laboratory-held populations regardless of the domestication duration. This suggests that thermal tolerance is maintained during domestication and highlights that experiments using domesticated laboratory-reared model species can be appropriate for addressing certain questions on thermal tolerance and global warming impacts.

## Teaser

Despite long-term domestication to stable temperatures, laboratory-strain zebrafish have kept the same upper thermal tolerance as wild-caught zebrafish held under laboratory conditions. Wild zebrafish tested in India, however, had a lower thermal tolerance highlighting that under certain conditions model organisms can be representative for climate change research.

## Introduction

Climate change is predicted to cause a continued increase in global water temperatures and increase the frequency and severity of extreme heat waves (Meehl and Tebaldi, 2004; IPCC, 2013; Seneviratne *et al.*, 2014). In some areas, this will impose stress on aquatic ectotherms as temperatures approach their thermal limits (Doney *et al.*, 2012; Sandblom *et al.*, 2016). Thermal stress triggers a multitude of physiological acclimation responses within the organism in order to improve overall function at non-optimal temperatures (Dietz and Somero, 1992; Angilletta, 2009). This phenotypic plasticity can be advantageous to organisms living in heterogeneous environments and could similarly be so under a climate change scenario. However, the thermal acclimation process can be both energetically costly (Angilletta, 2009) and come at a cost to fitness (Krebs and Loeschcke, 1994) and maintaining the capacity to acclimate when living in homogeneous environments, such as in a laboratory, might therefore be disadvantageous and selected against. This means that using laboratory model species for experiments on thermal tolerance might underestimate the acclimation potential of animals in nature. Yet, highly domesticated species are frequently used to answer questions on thermal tolerance and global warming impacts (Gilchrist *et al.*, 1997; Schaefer and Ryan, 2006; Paaijmans *et al.*, 2013).

Zebrafish (*Danio rerio*) are one of the most widely used laboratory species (Meyers, 2018). A wide range of domestication effects has been observed in zebrafish, both between laboratory-strain and wild-caught fish (Robison and Rowland, 2005; Whiteley *et al.*, 2011) as well in wild-caught zebrafish after being kept in the laboratory for only nine generations (Uusi-Heikkilä *et al.*, 2017). These domestication effects include behavioural (reduced startle response), physiological (increased growth rate) and genetic (genetic changes related to growth and maturation processes) alterations (Robison and Rowland, 2005; Uusi-Heikkilä *et al.*, 2017). Laboratory zebrafish are able to acclimate to a wide range of temperatures, and under normal holding temperatures (28°C), they have an upper acute thermal tolerance that exceeds 40°C (Cortemeglia and Beitinger, 2005; Schaefer and Ryan, 2006; Majhi and Das, 2013; Morgan *et al.*, 2018). This makes it a practical species to address questions within thermal biology, and it has previously been used in such experiments (reviewed in López-Olmeda and Sánchez-Vázquez, 2011). The zebrafish AB-WT laboratory population has been held in a controlled laboratory environment at optimal temperatures (26–28°C) with low thermal variation and few thermal extremes since the 1970s (Spence *et al.*, 2008). It could therefore be predicted that laboratory populations, through decades of adaptation to laboratory conditions with no adaptive value for high thermal tolerance, have lost some of their high temperature tolerance. Wild populations, on the other hand, experience daily and seasonally fluctuating temperatures with potentially harmful extreme temperatures (Engeszer *et al.*, 2007; Suriyampola *et al.*, 2016), suggesting that high thermal tolerance is of great adaptive value for wild zebrafish. However, whether there is a difference in thermal tolerance between laboratory-held lines, domesticated lines and wild zebrafish is currently unknown.

Surprisingly little is known about the natural environment of zebrafish, especially regarding their thermal background. Zebrafish inhabit rivers and streams in India, Nepal and Bangladesh and have been found in water temperatures ranging from 12.3–38.6°C (Engeszer *et al.*, 2007; Spence *et al.*, 2008; Arunachalam *et al.*, 2013). Wild zebrafish are therefore assumed to be tolerant to a wide temperature range and to have a high thermal tolerance. However, it is currently unknown what thermal safety margin (the difference between an organism’s thermal tolerance and the environmental extremes) wild zebrafish might have. As thermal extremes increase in frequency, the shallow waters inhabited by wild zebrafish may reach temperatures above their maximum thermal tolerance leading to mass mortality. Whether or not zebrafish will be able to cope with future climate warming will partly be determined by their capacity for thermal acclimation. A greater understanding of the thermal biology and acclimation capacity of zebrafish both in the wild and in the laboratory is therefore urgently needed.

The aims of this study were the following:

1) To test if the domestication process, with lack of thermal extremes and thermal fluctuations, has reduced thermal tolerance of laboratory-held zebrafish compared to wild zebrafish, and if so, how soon the difference appears in the domestication process (Experiment 1).
2) To compare the effects of daytime water temperature acclimation versus laboratory temperature acclimation on acute thermal tolerance of wild zebrafish tested on-site in India versus in the laboratory (Experiment 2).

In Experiment 1, we measured the acute thermal tolerance of wild-caught zebrafish on-site in India and at three stages of the domestication process in the laboratory: (i) wild-caught zebrafish acclimated to the laboratory (28°C) for 1 month, (ii) F_1_ generation of wild-caught zebrafish (short-term domesticated) and (iii) AB-WT laboratory-strain zebrafish (long-term domesticated). In Experiment 2, we measured the acute thermal tolerance on-site in India of wild zebrafish collected at a range of temperatures, as well as of fish from the F_1_ generation that were acclimated to a wide temperature range (10–34°C) in the laboratory for 30 days. This allowed us to compare the acclimation capacity of wild and short-term domesticated zebrafish. To measure thermal tolerance, we used the critical thermal maxima (CT_max_) test, which provides a quick and repeatable measure of acute thermal tolerance in zebrafish (Morgan *et al*., 2018).

## Materials and methods

### Animal collection and housing

**Figure 1:**
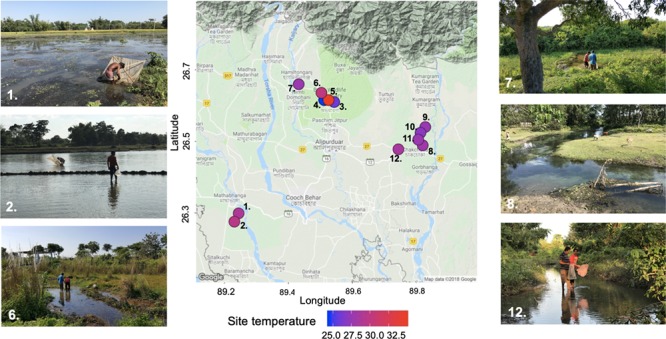
Locations of the 12 collection sites in West Bengal, India, visited in October 2016. The colour of each site represents the daytime water temperature measured in October 2016 (Table 1). Photographs show six of the sites (sites: 1, 2, 6, 7, 8 and 12; Table 1) where we collected both fish and environmental data.

Zebrafish were collected in North West Bengal, India, from 12 sites at the foothills of the Himalayas, close to India’s border with Bhutan and Bangladesh, in October 2016 (*n* = 4–10 fish per site; Fig. 1). All fish were collected by local fishermen using hand held nets in shallow water. Fish from 10 of the 12 sites (Fig. 1) were used in CT_max_ experiments performed on-site (described below).

Fish were also collected in August to September 2016 (*N* = 5000) from multiple sites in North West Bengal. These fish were imported by a commercial fish importer (Imazo AB, Sweden) and held in quarantine for 3 weeks in Sweden and subsequently transported to the laboratory aquarium facility at the Norwegian University of Science and Technology (NTNU), Trondheim, Norway, in November 2016. At NTNU, the ancestral zebrafish were housed in 63-l aquaria (maximum density, 0.5 fish l^−1^) with internal filtering and a flow-through system replacing 40% of the water volume daily. Temperature was maintained at 28 ± 0.5°C, and the water was well aerated. Fish were fed 0.1 g dry flakes (TetraPro) per aquarium twice per day and live *Artemia* sp. once per day. Tanks were cleaned as necessary. The F_1_ generation was produced by randomly grouping three males and three females of the ancestral generation together to breed multiple times. If fertilized eggs were found (after 14–20 hours), we knew that a minimum of 2 parents and a maximum of 6 parents had contributed to the F_1_ generation. In total, the F_1_ generation originated from a minimum of 350 ancestral parents.

AB-WT laboratory-strain zebrafish were obtained from the Yaksi laboratory at Kavli Institute, Trondheim, Norway, in October 2017. The fish were housed at NTNU under the same conditions as the wild-caught fish (in separate aquaria) before CT_max_ experiments commenced (see below).

### Experiment 1: effect of domestication on CT_max_

**Figure 2:**
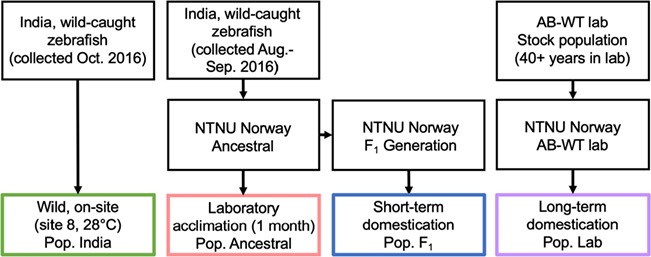
Graphical outline of Experiment 1 showing the origin of the fish (wild-caught collected in October and August to September 2016, AB-WT stock population), the location of the different experiments (on-site in India or at the NTNU) and the four different populations used (India, Ancestral, F_1_ and Lab). All fish in experiment 1 were acclimated to or had a site temperature of 28°C.

To investigate whether laboratory acclimation and/or domestication has an effect on thermal tolerance, we measured the CT_max_ of four populations of zebrafish. All populations were acclimated to, or had a site temperature of, 28°C: wild fish tested on-site (pop. India), laboratory-acclimated wild fish (pop. Ancestral), short-term domesticated wild fish (pop. F_1_) and long-term domesticated AB-WT laboratory strain (pop. Lab) (Fig. 2).

Pop. India: a randomly selected subset of the wild fish collected in October 2016 from site 8 (site temperature: 28°C, *n* = 10; Fig. 1) were used in on-site CT_max_ experiments (Fig. 2). The fish were kept in water and air-filled clear plastic fish bags, and their CT_max_ was measured approximately 6 hours after collection. During the ~ 6 hours holding period, fish were not fed.

Pop. Ancestral: CT_max_ of a randomly selected subset of the wild-caught fish (from the collection event in August to September 2016, *n* = 38) were measured when they had been held in stable laboratory conditions at 28°C at NTNU for 1 month (see housing conditions above, CT_max_ test performed in December 2016; Fig. 2).

Pop. F_1_: CT_max_ of short-term domesticated fish were measured using the F_1_ generation of the wild, ancestral fish (*n* = 10, kept for their entire life at 28°C, see housing conditions above and further details under Experiment 2 below, CT_max_ test performed in June 2017).

Pop. Lab: CT_max_ of long-term domesticated fish was measured using the AB-WT laboratory-strain zebrafish, which have been kept under controlled laboratory conditions (26–28°C) for more than 40 years. These fish are therefore the result of more than 70 generations (Howe *et al.*, 2013) kept at stable environmental conditions, which may have selected against a wide thermal tolerance (*n* = 20, experiment performed in February 2018 at NTNU; Fig. 2). All CT_max_ tests were performed according to the methods outlined below.

**Figure 3:**
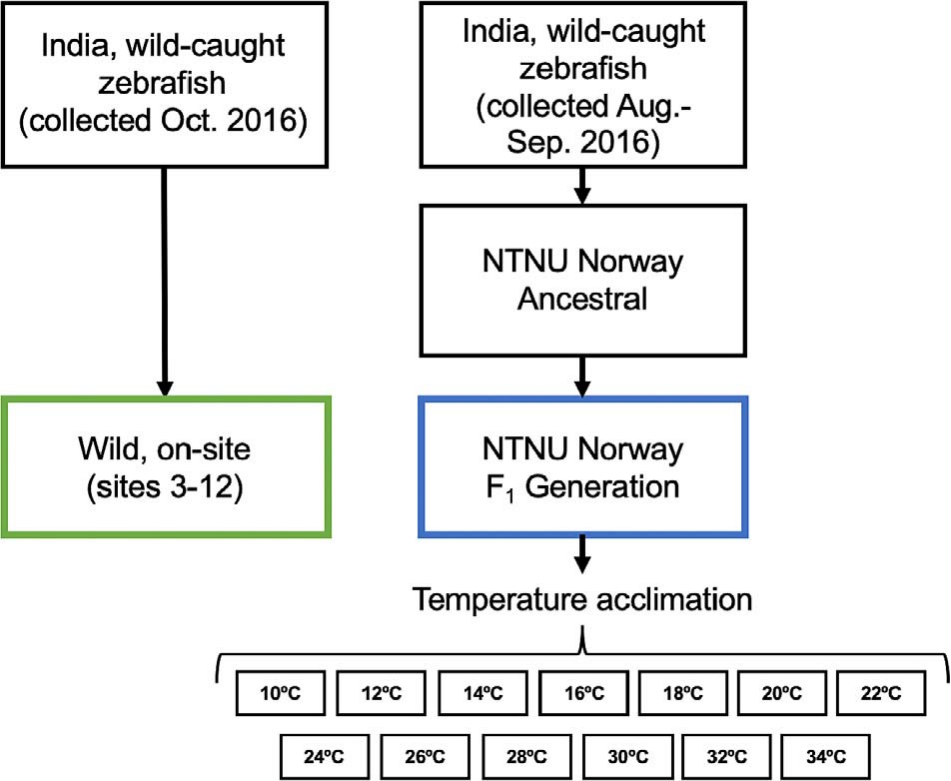
Graphical outline of Experiment 2 showing the origin of the fish (wild caught collected in October and August to September 2016), the temperature acclimation and the location of the different experiments (on-site in India or at the NTNU). The location of collection sites 3–12 (fish collected in October 2016) is shown in Fig. 1.

### Experiment 2: effect of temperature acclimation on CT_max_

To test whether laboratory-habituated and short-term domesticated wild fish (F_1_ generation) acclimated to a range of temperatures in the laboratory differ in their thermal tolerance compared to fish tested on-site in the wild (i.e. acclimated to a naturally varying range of temperatures), we conducted a temperature acclimation experiment at NTNU (in May–June 2017). A random subset of wild-caught zebrafish (F_1_ generation, *n* = 130, from the collection event in August to September 2016) were acclimated to temperatures ranging from 10–34°C in 2°C increments with 10 fish acclimated to each temperature (Fig. 3). Based on pilot experiments, these temperatures represent the range where zebrafish can maintain performance (i.e. swimming activity, feeding and growth). At the start of the experiment, all fish were 6 weeks old and housed at 28°C in 63-l aquaria, 10 fish per aquaria. In total, 13 aquaria were used, one allocated for each acclimation temperature (Fig. 3). The water temperature was increased or decreased in a daily step-wise manner (2°C per day) using titanium heaters (TH-100; Aqua Medic, Bissendorf, Germany) controlled by thermostats (ITC-306T; Inkbird, Shenzhen, China), until final acclimation temperatures were reached for all groups (after a maximum of 10 days). A group of 10 fish were constantly kept at 28°C, serving as the control (these fish were also included in the statistical analysis of experiment 1, as pop. F_1_). The fish were held for 36 days at these temperatures in closed system aquaria, with regular water changes. Temperatures were monitored in real time and continuously recorded (Picotech TC-08, Cambridgeshire, UK) in each aquarium. The fish were fed Tetra Pro flakes *ad libitum* twice daily. After 30 days, when the fish were 13 weeks old, all underwent a CT_max_ test, using the method outlined below.

The CT_max_ data from the F_1_ generation were compared to the CT_max_ data of wild fish tested on-site in India (Fig. 3). Randomly selected subsets of fish from 10 sites (Fig. 1: sites 3–12) of the collection event in October 2016 (*n* = 4–10 per site) were used as described above for Experiment 1 (where CT_max_ of fish from site 8 is described).

#### CT_max_ test

CT_max_ was measured using the method outlined in Morgan *et al*. (2018). In short, 4–10 zebrafish were placed inside a heating tank filled with 10 l water (with the corresponding temperature to the holding temperature of the fish). The water in the tank was continuously pumped over a coil heater, creating a homogenous heating rate of 0.3°C min^−1^ in accordance with Lutterschmidt and Hutchison (1997). Loss of equilibrium (LOE) was the CT_max_ endpoint (defined as the temperature at which disorganized and disorientated swimming occurred for at least 3 seconds). Upon onset of LOE, temperature was recorded to the nearest 0.1°C, and the fish was placed in 28°C water to allow recovery. Once the fish had recovered it was anaesthetized and weighed to the nearest 0.01 g. In all laboratory experiments, fish were fasted for 24 hours prior to the CT_max_ test and survival after the test was high (>95%). In the on-site experiments, the feeding state of the zebrafish was unknown, but the fish were not fed during the ~ 6 hours holding period prior to commencement of the CT_max_ test.

### Environmental data

During the collection event in October 2016, environmental data from 12 sites were collected (Fig. 1). We recorded the GPS coordinates and photographed the sites to document topography and vegetation coverage. Where possible, underwater video footage was taken of the fish at the field sites. Water temperature was measured using a high-precision thermometer (testo-112; Testo, Lenzkirch, Germany). Two water samples per site were collected to measure turbidity_NTU_ (nephelometric turbidity units, measured using a turbidity metre, HI93703; Hanna instruments, Limena, Italy). Conductivity was measured at each site (Jenway 4070, Stone, UK). Additionally, *p*CO_2_ was measured at seven sites (not all sites due to technical issues) [using a Vaisala GMT 222, (Vantaa, Finland) connected to a submerged gas-permeable PFTE probe (Qubit systems, Kingston, Canada), for details see Sundin and Jutfelt, 2016]. pH_NBS_ and O_2_ concentration were measured at one point on five of the sites (using a Hach Lange HQ40D multi-metre, Düsseldorf, Germany). The time of day when sampling occurred was recorded.

### Statistical analysis

Experiment 1: to investigate whether laboratory acclimation and/or domestication has an effect on thermal tolerance, we tested whether population (India, Ancestral, F_1_ and Lab) had an effect on CT_max_. We used an Analysis of covariance (ANCOVA) followed by a Tukey honest significance test (HSD) *post hoc* test, with CT_max_ as the response variable and population as the fixed effect. Weight was included in the model as a covariate. Since this analysis was performed on fish acclimated to a temperature of 28°C, and thus for the India population only included fish tested at one site (site 8, site temperature = 28°C) while the remaining populations originated from fish collected at several sites, we investigated whether any differences in CT_max_ could be due to differing genetic diversity by calculating the coefficient of variation (CV) for each population. We used Krishnamoorthy and Lee’s (2014) modified signed-likelihood ratio test from the R package cvequality (Marwick and Krishnamoorthy 2019) to compare the variability between populations.

Experiment 2: to investigate whether thermal acclimation capacity differs between wild and short-term domesticated fish, we used a subset of the temperature acclimated F_1_ generation fish (acclimation temperatures, 24–34°C) and compared the CT_max_ of those with the India population tested on-site across acclimation (i.e. site) temperature. The laboratory acclimation range of 24–34°C was chosen as it represents the range of site temperatures in India. A linear model was used to test the relationship between temperature and population on CT_max_. The interaction between temperature and population was also included in the model. A second-order polynomial regression was fitted to illustrate the effect of acclimation temperature on CT_max_ on the F_1_ generation across the whole temperature acclimation range (i.e. 10–34°C).

To investigate whether additional environment data affected on-site CT_max_ in India, a linear mixed effects model was fitted with CT_max_ as the response variable, site temperature and conductivity as the fixed effects and site number as a random effect (since multiple fish were measured per site). We also measured turbidity at the 10 sites (Table 1), however, due to the positive relationship between turbidity and conductivity (*r* = 0.62, *P* < 0.001), both could not be included in the same model. We chose to include conductivity over turbidity as the measurements of conductivity were more stable. Temperature and conductivity were not strongly correlated (*r* = 0.17, *P* = 0.13) and could therefore be included in the same model. Since *p*CO_2_, pH and oxygen saturation were not measured at all sites (Table 1), they could not be included in the model.

All analyses were done in R 3.4.3 (R Core Team, 2017) with effect sizes with *P*-values less than 0.05 considered statistically significant. Model assumptions (normality of residuals and homoscedasticity) were verified by visual inspections of residual-fit plots and q–q plots.

## Results

### Experiment 1: effects of laboratory acclimation and domestication on CT_max_

**Figure 4:**
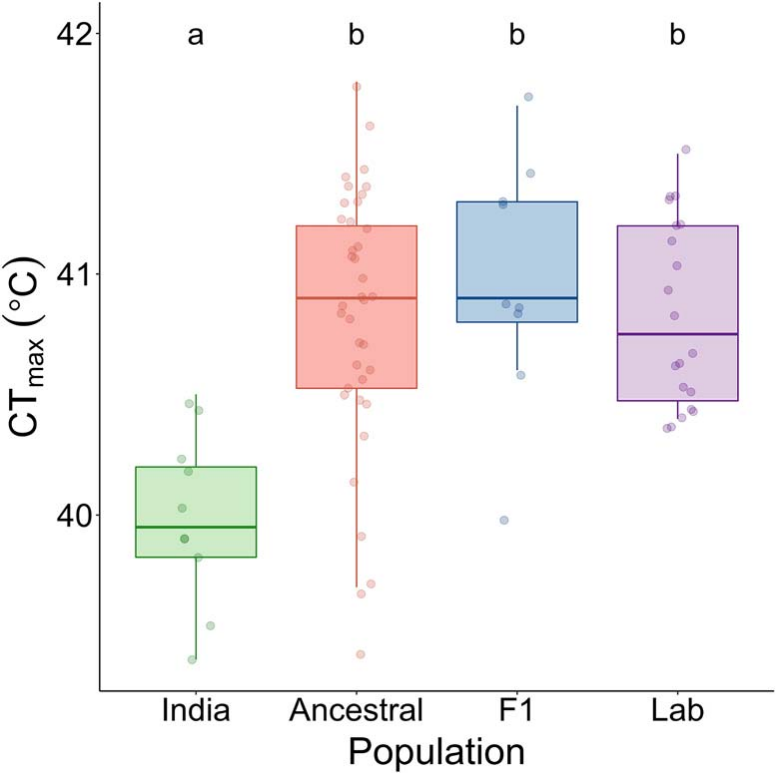
The effect of laboratory acclimation and short- and long-term domestication on thermal tolerance (CT_max_) in zebrafish. Comparison between wild fish tested on-site in India (India, site number 8, *n* = 10), laboratory-acclimated (1 month) wild fish (Ancestral, *n* = 38), the F_1_ generation of the wild fish (F_1_, *n* = 10) and the domesticated AB-WT laboratory-strain zebrafish (Lab, *n* = 20). Letters (a and b) indicate significant differences between populations. Raw data are shown as datapoints overlayed on the boxplots. All fish had been acclimated to, or had a site temperature of, 28°C prior to the CT_max_ test. The boxplots show first and third quartiles (boxes), the median (line in box) and the minimum and maximum values (whiskers, excluding outliers).

There was an effect of population on CT_max_ (*F*_3,72_ = 9.80, *P* < 0.0001; Fig. 4), with wild-caught zebrafish (measured at site 8 in India, site temperature = 28°C) having a lower CT_max_ than Ancestral (−0.85°C: *P* < 0.0001), F_1_ (−1.01°C, *P* = 0.0001) and Lab (−0.85°C, *P* = 0.0001: Fig. 4). Fish weight had no effect on CT_max_ (*F*_1,72_ = 2.48, *P* = 0.12). Individual variation in CT_max_ did not differ between the populations (test statistic = 4.50, *P* = 0.21, CV: Ancestral = 1.34, F_1_ generation = 1.23, India = 0.90 and Lab = 0.93).

### Experiment 2: effects of thermal acclimation on CT_max_

**Figure 5:**
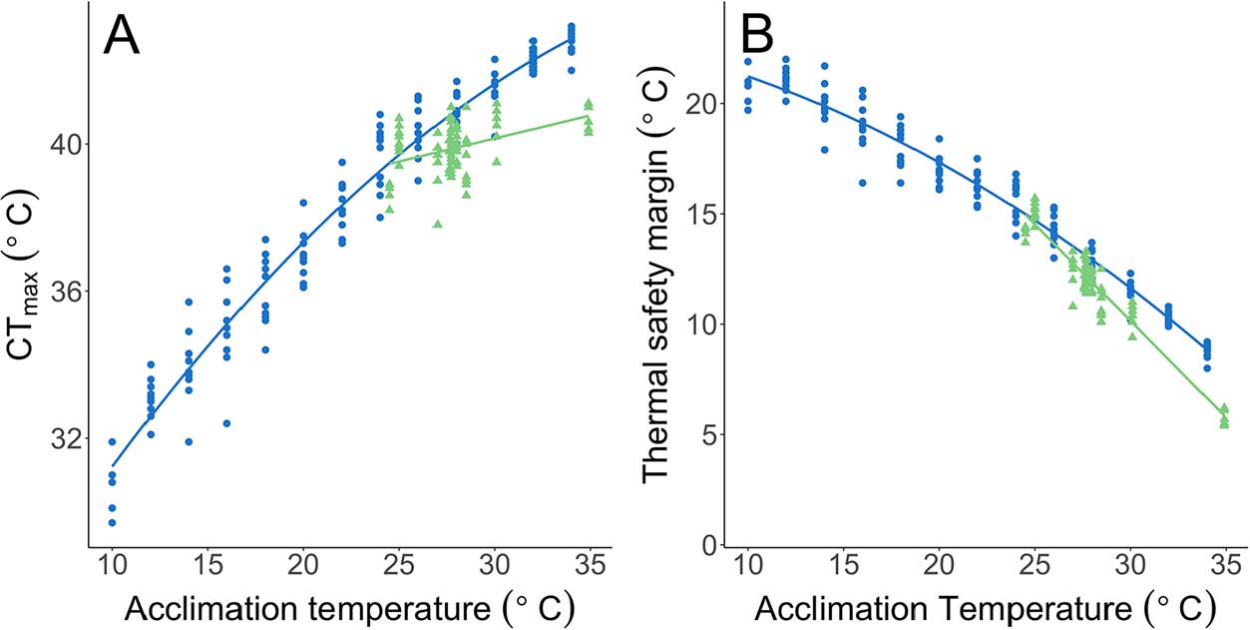
The relationship between site/acclimation temperature and (**A**) thermal tolerance (CT_max_) and (**B**) thermal safety margin (difference between acclimated temperature and thermal tolerance) of wild-caught zebrafish tested on-site in India (i.e. adapted to naturally varying field conditions) (*n* = 4–10 per site, green line and triangles) and fish acclimated to a temperature range under controlled laboratory settings after short-term domestication (F_1_ generation, *n* = 5–10 per acclimation temperature: blue line and circles).

Thermal tolerance did not differ between the F_1_ (39.67 ± 0.14°C) and the India (−0.27 ± 0.19°C, *P* = 0.162) populations at 24°C. However, acclimation temperature had a stronger effect on thermal tolerance (i.e. had a steeper slope) in the F_1_ generation (β = 0.32 ± 0.02) than the wild fish tested on-site in India (β = −0.19 ± 0.04). This is shown by the interaction between population and acclimation temperature (*F*_1,140_ = 27.92, *P* < 0.0001; Fig. 5A). The thermal safety margin (defined here as the difference between acclimation temperature and acute thermal tolerance) ranged from 5.4–15.7°C for the fish measured on-site in India and between 8.0 and 22.0°C for the F_1_ generation wild fish measured in the laboratory (Fig. 5B).

**Figure 6:**
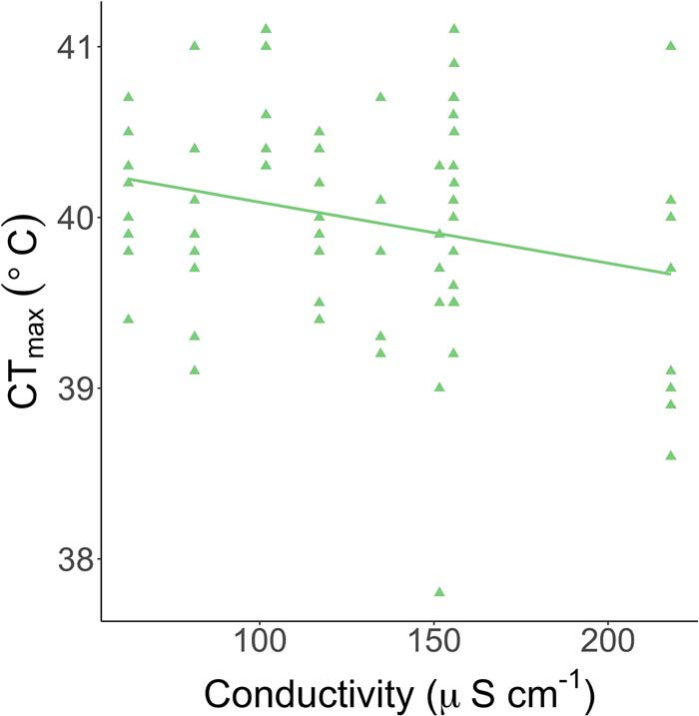
The relationship between thermal tolerance (CT_max_) and conductivity (μS cm^−1^) of wild-caught zebrafish at 10 sites in India. One conductivity measurement was taken per site, and thermal tolerance was tested in the zebrafish on-site (*n* = 4–10 fish per site).

When investigating if other environmental variables measured on-site in India affected CT_max_, aside from site temperature, we found a negative relationship between conductivity and CT_max_ (β = −0.0045 ± 0.001, *F*_1,78_ = 7.23, *P* = 0.04, Fig. 6) and only 4% of the variance was explained by site number. This negative relationship is, however, driven by the site with the highest conductivity (site 12; 218 μS cm^−1^) as there is no significant relationship if this site is removed (β = −0.0034 ± 0.001, *F*_1,70_ = 1.79, *P* = 0.24).

### Environmental data

Fish collection sites (collection event in October 2016) were clustered around 3 locations, sampled over 3 days (Table 1 and Fig. 1). The sites ranged from open riverbanks with clear flowing water to dense forests with turbid and slow-moving water (Fig. 1). Daytime site water temperatures ranged from 24.5–34.9°C (Table 1 and Fig. 1). The *p*CO_2_ measurements showed high levels of dissolved CO_2_ at all sites (Table 1), and therefore it is perhaps unsurprising that zebrafish behaviour is largely unaffected by elevated CO_2_ (Vossen *et al.*, 2016). Oxygen levels varied between sites (3.92–8.92 mg l^−1^; Table 1), and since zebrafish are hypoxia-tolerant species that are able to survive and grow at O_2_ levels down to 0.8 mg l^−1^ (van der Meer *et al.*, 2005), the levels measured here are comfortably within the species tolerance range.

## Discussion

Wild zebrafish tested on-site in India had a lower thermal tolerance (CT_max_) compared to short-term laboratory-acclimated wild zebrafish, first generation laboratory-reared wild zebrafish, as well as domesticated AB-WT laboratory zebrafish. The opposite result, i.e. that wild fish have a higher CT_max_ than domesticated lines, has previously been shown in certain species of trout, where it was suggested that genetic differences due to domestication could have altered thermal tolerance (Vincent, 1960; Carline and Machung, 2001). Indeed, daily thermal fluctuations and extreme temperatures, such as what many species of fish experience in the wild, have previously been shown to increase, not decrease, thermal tolerance both in zebrafish (Schaefer and Ryan, 2006) and in other ectotherms (Feldmeth *et al.*, 1974; Kern *et al.*, 2015). It is therefore somewhat surprising that we found the opposite result here. The improved conditions in a laboratory environment (e.g. high water quality and no diseases or parasites) could provide an explanation for the increased thermal tolerance in the laboratory. This was speculated to be the reason for a similar increase in thermal tolerance in wild-caught *Drosophila* after being brought into the laboratory (Krebs *et al.*, 2001). Diet is also known to modulate the thermal tolerance in fish (Hoar and Cottle, 1952; Gomez Isaza *et al.*, 2019), so the shift from a natural diet to laboratory diet (dry food and artemia) could have increased the thermal tolerance. The laboratory-held fish were fasted for 24 hours prior to testing their thermal tolerance, while the feeding state of the fish in the wild was unknown (but no food was provided during the 6 hours holding period before the CT_max_ test commenced). If the wild fish had recently fed, this could have reduced their CT_max_. In addition, the wild fish may have experienced greater handling stress than the laboratory-held fish and thus might have been more sensitive to the testing conditions. The site temperature in India was also only measured once at each site and during daytime. Therefore, the recorded temperature at the time of collection may represent the peak temperature and be an overestimation of the average daily temperature that the fish experienced and were acclimated to. However, since it was not possible to stay at each field site for a full day or return to the sites over different seasons we do not know the extent to which daily or seasonal temperatures fluctuated at these sites and the effect this could have on the wild zebrafish’s thermal tolerance. Finally, the lower thermal tolerance in the wild could be due to a low genetic diversity at each of the sites. At 28 degrees, the wild fish tested on-site came from just one site, compared to the ancestral and F_1_ populations that originated from multiple sites. However, we were unable to detect any differences in variation between the populations tested here.

**Table 1:** Environmental data from 12 collection sites in West Bengal, India, sampled in October 2016.

Site	Coordinates	Date	Time	Temperature (°C)	Turbidity_NTU_	Conductivity (μS cm^-1^)	*p*CO_2_	pH_NBS_	O_2_ (mg L^-1^)
1	26°17'56"N, 89°14'40"E	26-Oct	12:30	28.9	5.74, 5.88	122.4	-	-	-
2	26°16'32"N, 89°13'52"E	26-Oct	14:00	29.7	6.31, 16.40	97.6	-	-	-
3	26°36'36"N, 89°32'00"E	27-Oct	11:00	25.9	4.46, 4.47	62.3	4830	-	-
4	26°36'56"N, 89°31'32"E	27-Oct	-	24.5	-	-	-	-	-
5	26°36'56"N, 89°31'32"E	27-Oct	-	34.9	2.65, 6.52	101.8	-	-	-
6	26°38'09"N, 89°30'12"E	27-Oct	13:30	30.1	2.03, 2.62	155.8	4450	-	-
7	26°39'35"N, 89°25'56"E	27-Oct	14:30	27.7	3.77, 5.64	81.3	4730	-	-
8	26°29'21"N, 89°49'18"E	28-Oct	10:40	28.0	6.63, 17.90	117.1	4760	7.2	8.62
9	26°31'49"N, 89°49'39"E	28-Oct	12:15	27.6	2.92, 3.81	134.7	4700	7.4	8.86
10	26°31'27"N, 89°48'47"E	28-Oct	12:30	27.0	11.70, 12.80	151.6	4780	7.1	4.25
11	26°30'18"N, 89°48'21"E	28-Oct	13:00	27.7	4.60, 4.75	155.6	4730	7.6	8.92
12	26°28'41"N, 89°44'38"E	28-Oct	15:30	28.5	19.30, 20.90	218.0	-	7.3	3.92

Turbidity was measured in duplicate, data for both samples are reported

Counter to our prediction, there was no difference in thermal tolerance between the laboratory-acclimated wild-caught zebrafish and domesticated groups. The long-term domesticated line had the same, high, CT_max_ as both the ancestral laboratory-acclimated population and the F_1_ short-term domesticated population. This suggests that thermal tolerance is not a costly trait for zebrafish and that the domestication process may not have as strong effect on thermal tolerance as it may have on other traits. This is further supported by Stitt *et al.* (2014) who found that the thermal tolerance of brook trout (*Salvelinus fontinalis*) populations still reflect the historical adaptations to conditions in their ancestral sources despite being maintained for many generations in captivity. Furthermore, the population with the longest time in captivity (25 generations) had the highest thermal tolerance of all populations tested (Stitt *et al.*, 2014). Possible reasons for why thermal tolerance has been conserved in zebrafish in the absence of thermal stress could be related to a potentially complex genetic architecture underlying the trait. Currently, very little is known about the specific genes involved in thermal tolerance. As a wide range of processes (molecular, cellular and physiological) are thought to be able to affect thermal tolerance; however, it is likely that many genes would have to be modified to see an evolutionary change (Bettencourt *et al.*, 1999). In *Drosophila*, for example, a trade-off association between heat and cold tolerance has been identified suggesting the genes for these two traits are either highly linked or pleiotropic (Norry *et al.*, 2008). In addition, if genes for thermal tolerance are pleiotropic or highly linked to genes for other fitness traits that are under high selection pressure in the laboratory (e.g. fecundity and growth rate), this would further constrict evolvability and offers an additional explanation to why thermal tolerance has been conserved in the absence of thermal stress. Additionally, if evolution of thermal tolerance is a slow process, then zebrafish may not have been domesticated for a sufficient time.

High crowding densities and inbreeding are two conditions that are likely to occur during domestication (Lorenzen *et al.*, 2012). Both of these factors have been shown to increase the production and expression of heat-shock proteins (Hsps) (Sørensen and Loeschcke, 2001; Kristensen *et al.*, 2002; Sørensen *et al.*, 2003), and the expression of Hsps, in some cases, is correlated with an organism’s thermal tolerance (Iwama *et al.*, 1999). This suggests that Hsps could be under stabilizing selection in the laboratory and provides an alternative hypothesis to why high thermal tolerance has been maintained under optimal thermal conditions in laboratory-held fish. Importantly, we only used one of a multitude of available strains of laboratory zebrafish. We do not know whether all laboratory strains or even all populations within the AB-WT strain will have maintained their thermal tolerance during domestication or whether this is unique to the specific population that we tested.

Acclimation temperature is commonly known to affect an ectotherm’s thermal tolerance (Beitinger and Bennett, 2000; Schulte *et al.*, 2011; Huey *et al.*, 2012), and this is what we observed in zebrafish both in the wild and in the laboratory. Even though there was a positive relationship between water temperature and thermal tolerance in the wild, water temperature had a much stronger effect on thermal tolerance for laboratory-acclimated wild fish (F_1_ generation), suggesting the F_1_ generation differ in their acclimation capacity compared to the wild fish. In the wild, zebrafish spawn mostly at the onset of the monsoon season (Spence *et al.*, 2007) during which time water temperatures could be low. If the temperature during embryonic development was higher in the laboratory-reared F_1_ generation (26–28°C) compared to in the wild, this could explain the differences in acclimation capacity (Schaefer and Ryan, 2006). In addition, while the acclimation temperature was precise and constant for the F_1_ generation, it was an imprecise measurement in the wild, since the thermal profiles of the sites are unknown. It is therefore challenging to compare laboratory acclimation temperature and site temperature.

Despite these uncertainties, the thermal safety margin (difference between acclimated temperature and upper tolerance temperature) was just 5.4°C at the warmest site in India and decreased with increasing acclimation temperature. Since our measurements were not taken at the warmest time of the year it is likely that the maximum temperatures at these sites can be higher. Indeed, zebrafish have been found in the wild at temperatures as high as 38.6°C (Engeszer *et al.*, 2007). For fish living at the highest temperatures, this leaves only a very small thermal safety margin to buffer future warming events. Additionally, acute CT_max_ measurements tend to overestimate the thermal tolerance, and fish show lower tolerance temperatures during longer thermal exposures (Terblanche *et al.*, 2007). This could suggest that the thermal safety margin can be very low in wild zebrafish and that thermal tolerance is under strong selection pressure.

The high temperatures that zebrafish likely experience in the wild could provide an alternative explanation for why high thermal tolerance has been maintained in laboratory populations. If there has been strong selection on high thermal tolerance in the wild historically, then the genetic diversity for this trait could have been reduced (Falconer, 1981). Since genetic diversity is a prerequisite for evolution (Falconer, 1981), lack of genetic diversity would mean that the trait would not evolve or is fixed. A low genetic diversity in thermal tolerance in the founding population of the AB-WT laboratory fish could be the reason why high thermal tolerance persists in the absence of extreme temperatures despite decades of domestication.

In India, the conductivity of the water where we found zebrafish was lower (62.3–218.0 μS cm^−1^) than what is commonly used in zebrafish facilities (Avdesh *et al.*, 2012). It is surprising therefore that a relatively small increase in conductivity had a negative effect on thermal tolerance. However, a follow-up laboratory experiment (Åsheim *et al.*, in preparation) over a much wider range of salinities found that salinity had no effect on CT_max_ in zebrafish. This suggests that the negative relationship we found in India was caused by a confounding factor (e.g. pollutants, turbidity and nutrient load) that co-varies with conductivity rather than being caused by conductivity itself. This is further supported by the positive correlation between conductivity and turbidity that was found in this study.

In conclusion, we have shown that thermal tolerance is lower in wild zebrafish tested on-site compared to in laboratory-acclimated and domesticated populations. The lower thermal tolerance and reduced acclimation response observed in India was likely due to environmental rather than genetic effects, but it does illustrate that wild zebrafish can be found very close to their thermal limits. If the lower thermal tolerance observed in the wild compared to the laboratory was due to some of the reasons discussed (e.g. diet, disease and stress) then we might overestimate the warming tolerance of wild populations if using laboratory experiments alone, and this does provide some cause for concern. Time in captivity, however, had no effect as CT_max_ did not differ between short-term laboratory-acclimated and domesticated fish. Thermal tolerance therefore appears to be a robust trait, which has been maintained during domestication in zebrafish. Experiments using laboratory-kept species and model organisms may therefore sometimes be appropriate for addressing questions about the impacts of global warming.

## Ethics

The experiments were approved by the Norwegian Animal Research Authority (permit number: 8578), and all methods were performed in accordance with the relevant guidelines and regulations. The fieldwork performed in India, including capture and handling of zebrafish, was covered under permit number: 1932802178001, obtained from the Directorate of Fisheries, Government of West Bengal. In accordance with the Indian government’s Biodiversity Act, no fish or biological samples were brought out of India by the researchers.
